# Diagnosis and Treatment of Adrenocortical Oncocytoma: Case Report of Five Cases and Review of the Literature

**DOI:** 10.3389/fonc.2019.00338

**Published:** 2019-05-02

**Authors:** Dexin Dong, Xiao Liu, Zhigang Ji, Hanzhong Li

**Affiliations:** Department of Urology, Chinese Academy of Medical Sciences, Peking Union Medical College Hospital, Peking Union Medical College, Beijing, China

**Keywords:** adrenocortical oncocytoma, treatment, endocrine examination, pathology, surgery

## Abstract

**Objective:** To investigate the diagnosis and treatment of adrenocortical oncocytoma, and have a literature of review.

**Materials and Methods:** The clinical data of 5 cases of adrenocortical oncocytoma treated in our hospital was retrospectively analyzed. The clinical manifestations, imaging examination, endocrine examination, and pathological results were analyzed respectively.

**Results:** Oncocytic adrenocortical neoplasms are extremely rare. Oncocytic adrenocortical neoplasms are usually discovered incidentally, only the tumors with endocrine function could exhibit specific manifestations. No specific imageological features of oncocytic adrenocortical neoplasms have been found.

**Conclusions:** The diagnosis of adrenocortical oncocytoma mainly depends on the pathological examination. Surgical resection is the main treatment method.

## Introduction

The oncocytic adrenocortical neoplasm is a rare tumor of the adrenal gland. Since it was first reported in 1986 (1), there has been serial case reports to try to illuminate this rare tumor. However, there is still rather little information available, especially the pathological and follow-up data, to illustrate the biological behavior of this particular tumor. The origin, biological behavior, diagnostic criteria, and prognosis of oncocytic adrenocortical neoplasm remain controversial. Here, we report 5 cases with oncocytic adrenocortical neoplasm and try to summarize its clinical and pathological manifestations, diagnostic criteria, surgical treatment, and prognosis. Written informed consent was obtained from the participant for the publication of this case report and any potentially-identifying information/images.

## Materials and Methods

The demographic and clinical data of 5 patients with oncocytic adrenocortical neoplasms, diagnosed and surgical treated at the department of urology in Peking Union College Hospital, between April 2005 and July 2018, were retrospectively analyzed.

As showed in Table 1, there were 1 male and 4 females. The age ranged from 17 to 63 y. Only one patient found the tumor incidentally during the healthy examination, 2 cases discovered the tumor during body check for none specific symptoms of palpation or flank pain and the other 2 cases were diagnosed the adrenal tumor during evaluation of the Cushing syndrome, whose urinary free cortisol were significantly elevated. Case 4 underwent resection of left adrenal oncocytoma (10^*^8^*^6cm) 6 years ago. She felt flank pain before abdominal CT confirmed the recurrence of tumor near the left kidney and in the abdominal wall. Case 5 manifested virilization besides Cushing syndrome, such as hairy face, rough skin, and irregular menstruation, whose serum testosterone level rose. The cortisol, aldosterone, and catecholamine metabolites were normal except for Cushing Syndrome cases. Except case 1, the tumor size of the other 4 cases were more than 6 cm. Three cases underwent laparoscopic adrenal tumor resection, and 2 patients underwent open surgery of adrenal tumors. All procedures were successfully performed and no complications occurred. The tumor specimens were carefully examined and pathological report indicated adrenocortical oncocytoma in all cases, 2 of which were uncertain malignant potential according the Lin-Weiss-Bisceglia system (2). All the patients were regularly followed up. The follow-up ranged from 7 to 154 months. The virilization and Cushing syndrome disappeared and the serum cortisol and testosterone returned to the normal during the follow-up. There were no local recurrence and distant metastases in all cases.

**Table 1:** Clinical information of the patients.

				**Hormones**									**Pathology**									
**Case**	**Age(y)**	**Gender**	**Symptoms**	**24h UFC**	**NE**	**E**	**DA**	**T**	**DS**	**Ald**	**Surgical** **approach**	**Tumor** **size**	**Tumor** **site**	**Tumor** **weight**	**Malignant**	**Melan-A**	**Synaptophysin**	**α** **-inhibin**	**Calretinin**	**Vimentin**	**Ki-67**	**Follow-up**
1	23	F	Cushing syndrome	218.1	15	2.68	237.6	–	–	13.7	Laparoscopic	3*2.5*2	Left	11	Benign	None	None	None	None	None	None	154
2	63	F	Palpitation	116.6	18.51	1.85	187.92	–	–	11.5	Laparoscopic	7*6.5*4	Left	76	Benign	None	None	None	None	None	None	81
3	50	M	None	85.93	21.69	3.56	225.69	–	–	15.1	Laparoscopic	6*6*5.5	Left	127.1	UMP	+	+	–	+/–	+	10%	30
4	55	F	Flank pain, post-op of left adrenal oncocytoma for 6 years	36.96	–	–	–	–	–	–	Open surgical	8.8*7.8*7, 2.6*2.1*1.5	Left kidney, abdominal wall	–	benign	–	+	–	+	+	1%	19
5	17	F	Virilization, Cushing syndrome	265.76	17.72	1.9	159.87	2.55	1291.9	23.54	Open surgical	10*7*6	Right	183.6	UMP	+	+	+	+	–	3%	7

*(UMP uncertain malignant potential). 24 h UFC, 24 h urinary free cortisol; NE, Norepinephrine; E, epinephrine; DA, Dopamine; T, testosterone; DS, Dihydrotestosterone; Ald, aldosterone*.

**Figure 1:**
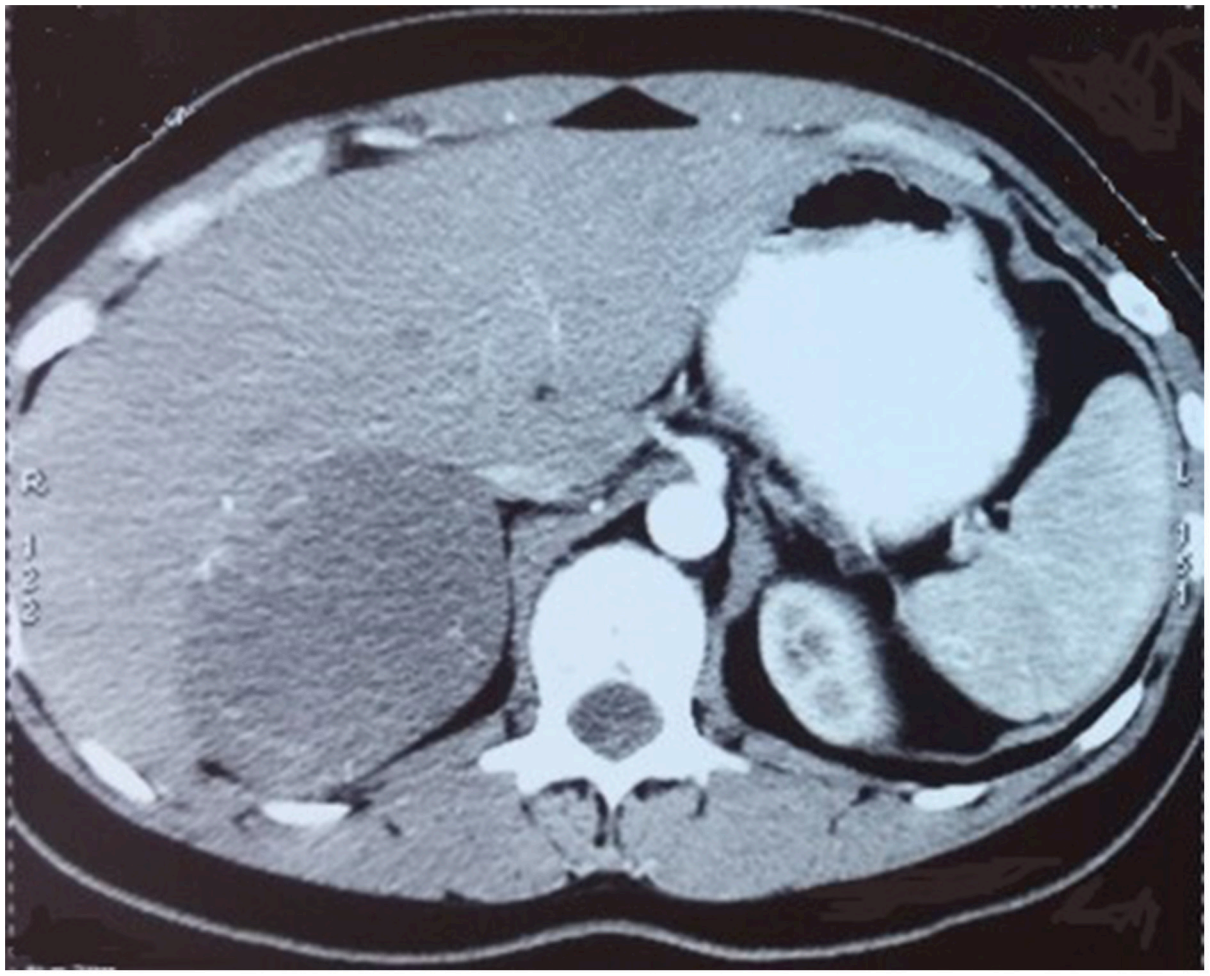
CT scan showed a round mass between liver and right kidney with heterogeneous enhancement.

**Figure 2:**
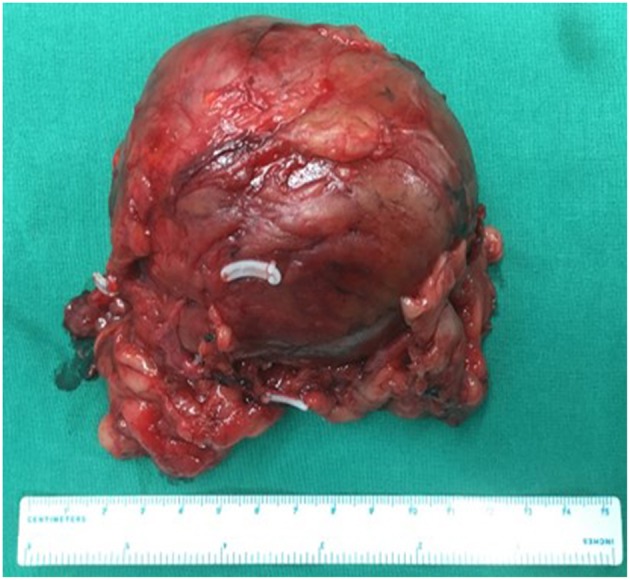
Adrenal oncocytoma is a rounded and encapsulated mass (10*7*6 cm), with yellow-brown cut section. Microscopically, tumor cells are highly eosinophilic and arranged in a solid pattern.

**Figure 3:**
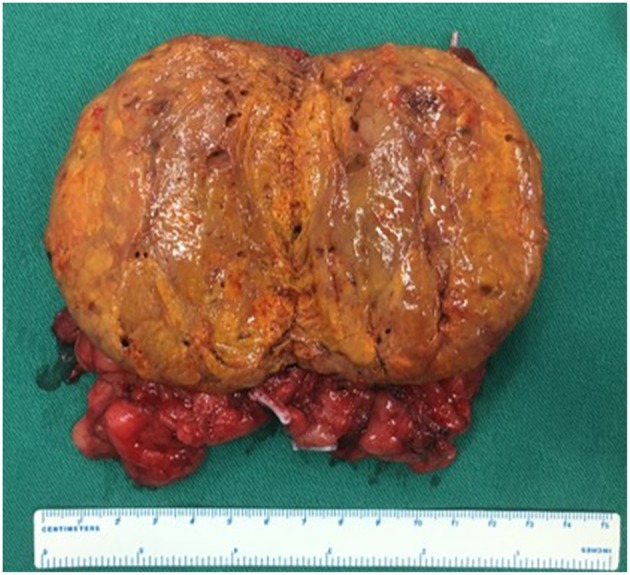
Same as Figure 2.

**Figure 4:**
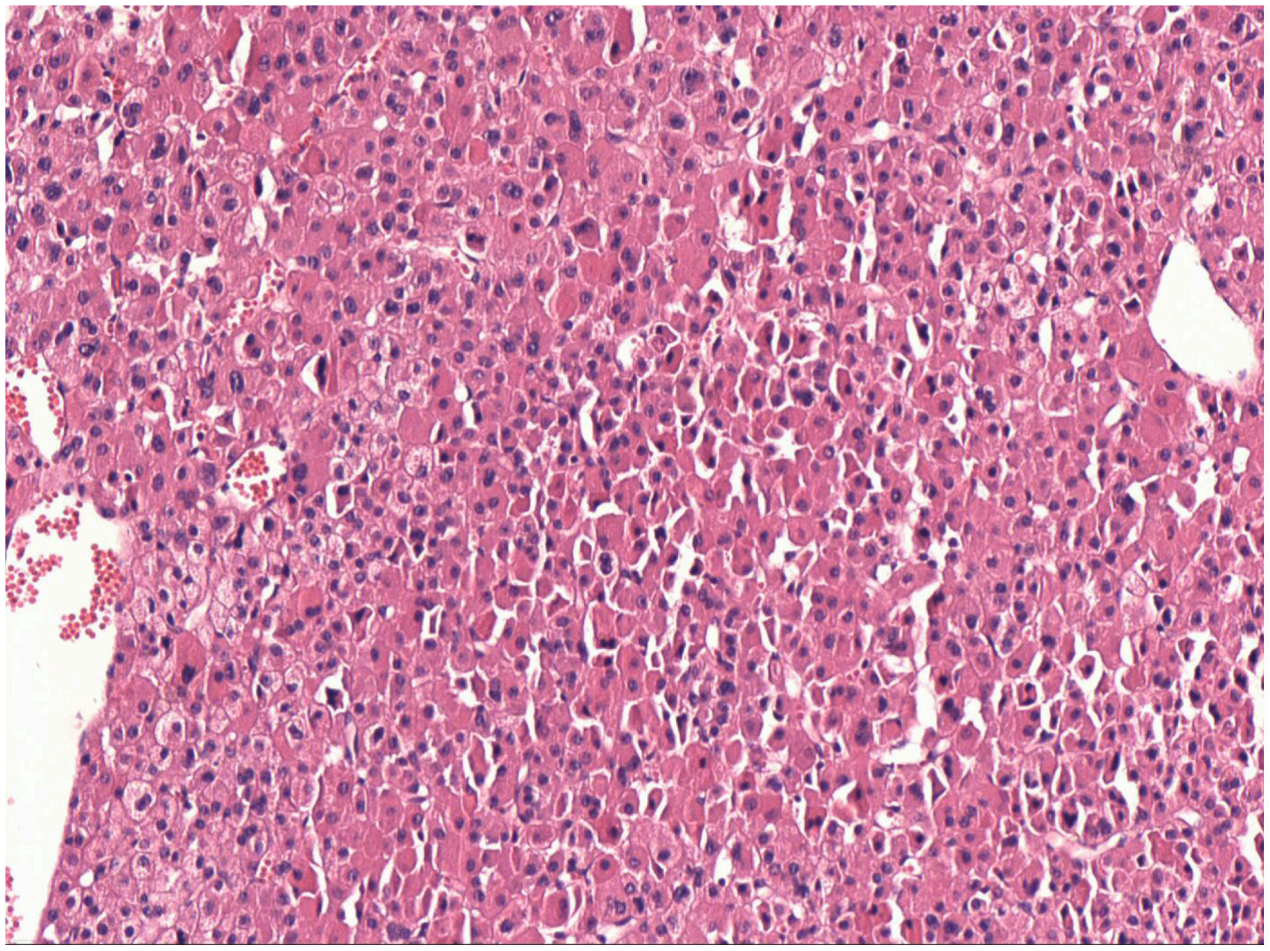
Same as Figure 2.

The characteristics of the patients were showed in Table 1. The CT scan of case 5 showed the tumor located between the liver and kidney without normal adrenal gland left. The enhancement was heterogeneous (Figure 1). The tumor of case 5 was rounded and encapsulated, whose cut section was yellow-brown. HE staining showed the tumor cells were highly eosinophilic and arranged in a solid pattern (Figures 2–4). The study is approved by institutional review board of Peking Union Medical College Hospital.

## Discussion

Oncocytic neoplasms are tumors where granular eosinophilic cytoplasmic cells resulting from accumulation of mitochondria are the dominant cell type. They are mostly benign tumors and usually arising in the kidney, salivary gland, and pituitary. But according to a series of case reports, it seems that oncocytic neoplasms could originate from any organ, including adrenal gland, thyroid, parathyroid gland, thymus, stomach, liver, pancreas, breast, upper respiratory tract, and so on. (3, 4) The oncocytic neoplasms may share similar molecular alterations and biological features despite originating from different organs (5, 6).

Oncocytic adrenocortical neoplasms are extremely rare. There has been nearly 200 cases since this rare disease was first reported in 1986 (1). Adrenocortical oncocytomas could happen in a large age range, from adolescent to elderly people. This disease has a female dominance of about 2.5:1 and a left-side dominance of about 3.5:1 (4, 7). Our results are consistent with the literature. The age ranged from 17 to 63 years old. 4/5 of the cases were female and 4/5 of the tumors located at the left side.

Oncocytic adrenocortical neoplasms are usually discovered incidentally during routine body check without clinical manifestations (8). Only minority of patients may have non-specific symptoms such as abdominal pain, nausea, hypertension and so on (9, 10). Only the tumors with endocrine function could exhibit specific manifestations, such as virilization, feminization, and Cushing syndrome, while most oncocytic adrenocortical neoplasms show no function. There were also studies showed that nearly 30% of oncocytic adrenocortical neoplasms were functional (7). In this study, only one patient showed no symptom. Two patients had non-specific symptoms including abdominal pain and palpitation. And another two patients showed virilization and Cushing syndrome. So oncocytic adrenocortical neoplasms might be functional tumors.

No specific imageological features of oncocytic adrenocortical neoplasms have been found. Benign oncocytic adrenocortical neoplasms may be distinguishable from lipid-rich but not lipid -poor adenomas on CT examination. Malignant ones demonstrate similar features with adrenocortical carcinomas, such as large size, necrosis, and lower percentage enhancement washout, which makes differentiation through CT very difficult. So there were no CT or MRI criteria available to help differentiate benign from malignant tumors (11). There are also no specific signs in MRI and ultrasound (4, 12). Then the imaging examination is mainly used to confirm the location of the tumor and useless to differentiate benign or malignant. In this series, only case 1 exhibited homogeneous enhancement and the other 4 cases were heterogeneous enhancement.

The diagnosis of adrenocortical oncocytoma mainly depends on the pathological examination. Fine-needle aspiration cytology seems to be useful to confirm the diagnosis preoperatively, but because of the large tumor size and possible heterogeneous areas, this technique may not characterize the tumor and increase the risk of needle tract implantation metastases in case of malignancy (13, 14). The section of benign tumors is usually golden or brownish yellow and the malignant tumors are mostly ashes red or fish-meat like (12). The Weiss system has been adopted as the standard criteria for the assessment and categorization of adrenocortical neoplasms (15). But because of the lack of reported cases of oncocytic neoplasms of the adrenal gland and the follow-up data, Lin-Weiss-Bisceglia system was put up to revise the former criteria to help diagnose this unique tumor (16). The major criteria include mitotic rate more than 5 mitotic figures per 50 high-power fields, atypical mitoses and venous invasion. The minor criteria include large tumor (>10 cm and/or >200 g), necrosis, capsular invasion, and sinusoidal invasion. Presence of any major criteria would be diagnosed as malignant and presence of any minor criteria would be diagnosed as borderline or uncertain malignant potential, while presence of none of major or minor criteria would be diagnosed as benign (2). In our series, 3 cases were benign and the other 2 were uncertain malignant potential according to the modified Weiss system. The case 3 is special, which seems to be malignant as the tumor recurred in the left kidney and abdominal wall after 6 years of resection of left adrenal oncocytoma. There has been 19 months after the second surgery and no recurrence was found. The detailed biological behavior of this tumor remains unknown.

The therapy of oncocytic adrenocortical neoplasms mainly relies on the surgical resection. With the development of laparoscopic technique, the laparoscopic surgery is becoming more and more popular (17, 18). It was suggested to perform the laparotomy when the tumor size was more than 6 cm to obtain a complete resection without tumor rupture. In this study, 2 cases with tumor size more than 6 cm were performed laparoscopic surgery and there is no recurrence after 30 and 81 months follow-up. For the metastatic tumors, surgery is recommended if the metastasis is restricted and could be safely resected (19, 20). In this study, after complete resection of the recurrent tumors, the case 4 has a very good prognosis with no recurrence for nearly 1.5 years.

The oncocytic adrenocortical neoplasms are rare and mostly benign tumors. Surgical resection is the main treatment method. Careful pathological examination and close follow-up are needed to confirm the prognosis.

## Ethics Statement

The study is approved by institutional review board of Peking Union Medical College Hospital. Written informed consent was obtained from the participant for the publication of this case report and any potentially-identifying information/images.

## Author Contributions

DD and XL write the manuscript. ZJ revise the manuscript. HL review the manuscript.

### Conflict of Interest Statement

The authors declare that the research was conducted in the absence of any commercial or financial relationships that could be construed as a potential conflict of interest.

## Abbreviations

CT: computed tomography
MRI: magnetic resonance imaging.
